# The Use of Thermal Imaging in the Evaluation of the Symmetry of Muscle Activity in Various Types of Exercises (Symmetrical and Asymmetrical)

**DOI:** 10.1515/hukin-2015-0116

**Published:** 2015-12-30

**Authors:** Monika Chudecka, Anna Lubkowska, Katarzyna Leźnicka, Krzysztof Krupecki

**Affiliations:** 1 Department of Human Functional Anatomy and Biometry, Faculty of Physical Culture and Health Promotion, Szczecin University, Szczecin, Poland; 2 Department of Functional Diagnostics and Physical Medicine, Faculty of Health Sciences, Pomeranian Medical University in Szczecin, Szczecin, Poland; 3 Department of Theory and Practice of Sport, Faculty of Physical Culture and Health Promotion, Szczecin University, Szczecin, Poland

**Keywords:** thermal imaging, anthropometry, symmetry of muscle activity, coaching

## Abstract

In order to achieve higher efficiency of training and thus better athletic performance, new research and diagnostic methods are constantly being developed, particularly those that are non-invasive. One such a method is thermography, suitable for quantitative and therefore objective evaluation of variables, such as changes in the temperature of the skin covering working muscles. The aim of this study was to use a thermal imaging infrared camera to evaluate temperature changes of symmetric body surfaces over symmetrically working muscles of male scullers after exercising on a two-oared rowing ergometer and compare these to asymmetrically working muscles of handball players after an endurance training session containing elements of an actual game. In the scullers, the mean temperature of body surfaces was always lower post than pre exercise, with no significant differences in an average temperature drop between the opposite sides, indicating that the work of the muscles involved in the physical exertion on the rowing ergometer was symmetrical. In contrast, in the handball players, skin temperatures in symmetric areas over the asymmetrically working muscles showed statistically significant differences between sides, which was associated with the functional asymmetry of training. This study indicates that thermal imaging may be useful for coaches in the evaluation of technical preparations in sports in which equal involvement of symmetric muscles is a condition of success, e.g. in scullers.

## Introduction

The success in modern sports depends on rational training, brought to perfection by coaches and researchers collaborating with athletes in the training process. The optimal use of the abilities of athletes in training and competition requires the evaluation of the effectiveness of stimuli, and constant monitoring of training effects in relation to completed tasks. The decisive factor is data on the athlete’s functional potential and his/her ability to use it in an optimal way (Bernstein, 1988). This means that sport performance is the sum of tactical and technical skills, motor and coordination skills, as well as optimization of their development.

A rise in expertise in rowing sports in recent years has resulted in the increasingly detailed studies on rowing technique. Rowing is a sport in which the body moves in a closed cyclical manner. The rowing cycle consists of relatively independent periods with and without resistance. Each period is governed by specific goals for the sculler and the movement of the boat, along with spatial and temporal indicators dependent on the individual characteristics of a sculler and the type of boat being used. It is of immense importance for two-oared scullers that the muscles are equally involved in the performed movement, to achieve maximum velocity of the boat and an ideal course in the water.

In order to achieve higher training efficiency, and thus better athletic performance, new research and diagnostic methods are constantly being developed, particularly those that are non-invasive. One such method is thermography, which is suited for the quantitative and therefore objective evaluation of changes in the temperature of body surfaces covering active muscles (Akimov and Son’kin, 2011; Chudecka and Lubkowska, 2010, 2012). Non-contact thermography enables diagnosis of the distribution of skin surface temperatures from athletic movement (Fröhlich et al., 2013). Monitoring muscle activity by means of thermovision is possible, as shown by a correlation between performed mechanical work and the metabolic processes that produce heat (Anwajler and Dudek, 2009).

Thermal imaging techniques that measure body surface temperatures may be used to assess the degree of involvement of selected muscle groups during exercise. They can therefore be used as a tool for coaches to evaluate not only the dynamics underlying body surface temperatures from exercise, but also to assess the symmetry of muscle activity. In available literature, however, there are only a few papers that mention the use of thermography for the aforementioned purposes (Awrejcewicz and Zagrodny, 2011; Awrejcewicz 2012; Parkin et al., 2001; Vardasca et al., 2012). Our research indicates the potential usefulness of thermal imaging in the evaluation of the distribution of skin surface temperatures from exercise, especially in athletes whose success depends on symmetrical muscle activity.

The aim of this study was to use a thermal imaging infrared camera to evaluate temperature changes of symmetric body surfaces over the symmetrically working muscles of scullers after exercising on a two-oared rowing ergometer and compare these to the asymmetrically working muscles of handball players after endurance training containing elements of an actual game. Another aim of the study was to show the possibility of using this method to improve monitoring of training progress.

## Material and Methods

### Participants

The study involved two groups of male athletes: scullers and handball players, who represented sport disciplines with a very different nature of physical activity. Training and muscle activity of scullers is symmetric, in contrast to asymmetric training of handball players – set plays are performed with the dominant hand, so functional asymmetry has great significance.

The group of scullers included 18 male athletes from the university rowing club, average age of 20.77 years (SD=1.78), professionally involved in two-oared sculling. The study was performed in the precompetitive period. The average scullers’ training experience was 5.44 years (SD=1.77). The number of training sessions varied from 5 to 6 per week. The second study group comprised 16 professional handball players, average age of 22.54 years (SD=2.01), from a local 1st division team. The handball players’ average training experience was 8.31 years (SD=2.15). They trained 6 to 8 times per week.

Each athlete provided written consent before participating in the research, according to the Declaration of Helsinki. The study was approved by the Pomeranian Medical University Ethics Committee, (KB-0012/151/12). All participants were healthy and not injured.

### Procedures

The study was conducted during a regular training session, after a 30 min warm-up. Indexes of the resting heart rate were monitored by a Polar sport tester.

After recovery of the heart rate to resting values, the scullers were subjected to a maximal exercise on a Concept II two-oared rowing ergometer simulating a distance of 2000 m. This is a basic test used in rowing to reflect the conditions of a rower, and is also used in competitions in hall rowing. The data obtained during these tests provided information on exercise intensity, the heart rate and the pace of rowing. Duration of the exercise test on the Concept II in the group of scullers averaged 6 min (5.32–6.17), with the stroke rate in the range 28–32 (M= 30) strokes per min. During the exercise they achieved a heart rate of 190 (185–201) bpm, corresponding to a maximal effort for these rowers.

The handball players were tested during an endurance training session that contained elements of an actual game. Training lasted 90 min, during which the players’ heart rates were monitored by means of Polar sport testers, and then individual mean training heart rates (HRt) were determined. The authors also estimated individual percentage ratios of the mean heart rate at training to the maximum heart rate ( $\frac{HRt}{HRmax}\%$), which allowed defining the intensity of effort during the training session, which ranged from 71.96 to 82.35% (M=75.77%; SD= 3.202).

The rooms in which the training sessions took place had similar conditions; the air temperature was 20°C and air humidity was 55%.

**Figure 1:**
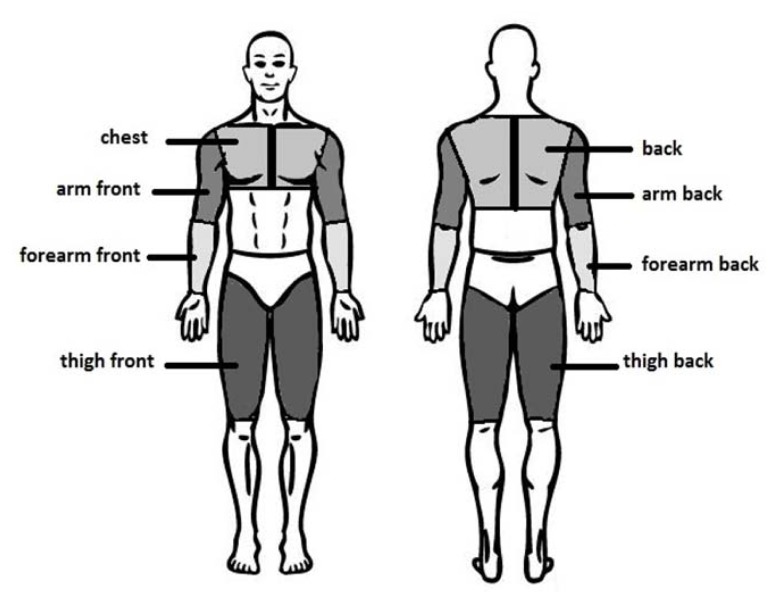
Analyzed body surfaces

For each participant, two series of thermograms were performed on selected body surfaces in a standing position: the front and rear surfaces of the upper limbs (arm and forearm), the chest, front and rear surfaces of the thighs, and the back. The analyzed areas are presented in Figure 1.

The study used a ThermaCAM SC500 camera (FLIR Systems Inc., Wilsonville, OR, USA). Two measurements were taken, at 25°C and humidity of 60% at a distance of 3 m. The first 20 min before training and the second following training. During the measurements the subjects were dressed in shorts and sport shoes to expose the selected surfaces. The thermography technique allows recording of skin temperatures and speed of temperature change to a resolution of <0.1°C. The following software was used for measurement analysis: Agema Report 5.4.1, Agema Report Viewer 5.4, and Agema Image Viewer 1.02 (AGEMA Infrared Systems, Sweden). The aforementioned series were as follows:

1. in the scullers group:
  Series 1 – before the training session (adaptation time: 30 min);
  Series 2 – directly after maximal physical exertion on the Concept II rowing ergometer.
2. in the handball players group:
  Series 1 – before the training session (adaptation time: 20 min);
  Series 2 – directly following 90 min of endurance training that contained elements of an actual game.

Duration of exercise and thermal imaging recording were identical for all the participants. The subjects began and finished training one after the other, so the time duration between exercise and testing time was the same for each participant. All participants were right-handed, apart from one handball player who was left-handed (it was included in the analysis and recorded as the dominant limb).

The selected surface areas were continuously exposed during the exercises. Mean temperature (T_mean_) within the selected areas was recorded. Computerized image analysis allowed selection of the measurement area on the thermograms. The tests were performed in accordance with the standards of the European Association of Thermology (Fuijmasa, 1995). Skin emissivity was assumed to be 0.98.

### Statistical analysis

Statistical characteristics of the examined variables are presented using arithmetic means and standard deviations. Data were found to be normally distributed (Kolmogorov-Smirnov test). To assess the significance of changes in mean temperatures ΔT_mean_ (before and immediately after the exercise) between the analyzed symmetric body surfaces, we used the Student t-test with statistical significance of *p*<0.05. Calculations were performed using Statistica 10 software (StatSoft, Inc., Tulsa, OK, USA).

## Results

The scullers’ average body height equaled 184.11 cm (SD=4.56) and body mass 82.33 kg (SD=7.19). The average handball players’ body height was 187.25 cm (SD=4.62) and body mass 86.27 kg (SD=7.17).

On the basis of the thermograms, changes in T_mean_ were recorded in the studied areas. Immediately after the exercise mean temperatures were always lower than before in all the subjects. The decreases in temperature in the analyzed body areas between the series of tests are shown in Tables 1 and 2.

In the scullers group (Table 1) there were no statistically significant differences between the mean drops in temperature of the compared symmetric areas over the groups of muscles active during the tests, with differences in the mean temperature drop less than 0.5°C between the opposite sides of the symmetrical areas. Moreover, the Student’s t-tests showed that there were no significant differences between temperature drops on the opposite sides in the scullers.

In contrast to this and as expected, in the handball players there were significant differences (Table 2) in the changes of temperature between the opposing symmetric body areas over the muscles involved in training. Differences were statistically significant for the areas of the right and left arm, and the left and right forearm (front and back). The Student’s t-tests showed significant differences in temperature drops between these opposite sides.

## Discussion

Working muscles and their increased metabolism cause a pronounced increase in core body temperature. The generated heat is received by the flowing blood and is transferred to the surfaces of the body, where it is lost. The most effective way of removing heat is through evaporation of sweat from body surfaces, which is why immediately after maximal exercise the body surface had a lower temperature than before the exercise (Schlader, 2010).

In a better-trained body, adaptive changes result in a smaller internal temperature increase and greater intensity of sweating. Consequently, cooling of the body is more effective and at the same time the surface temperature of the body after physical exertion is lower than in untrained subjects (Aoyagi, 1997; Smorawiński and Grucza, 1994; Torii, 1992). Thus, changes in the surface temperature of the body can be an indicator of the load of the locomotor system, providing information on the efficiency of systems responsible for removing endogenous heat generated during exercise.

This study does not focus on measuring the absolute temperature changes on the skin surface of an athlete participating in different kinds of physical exercise. We did not compare changes in the response to physical exercise, as the intensity of muscle activity was known to be distinctly different. Instead, the aim was to compare such responses between physical activities that involved muscle groups used in a decidedly symmetric or asymmetric manner. Correspondingly, we chose the groups of scullers and handball players due to their specific lateralization. The exercises performed by the scullers were general and required symmetry in the activity of muscles, while in handball players, the involvement and activity of the muscles were distinctly asymmetric.

During exercise, changes in mean temperatures of symmetrical body surfaces directly above the muscles engaged in physical exertion should be similar, as it is where body heat is generated. This excess heat can only be dissipated by increasing blood circulation in the skin, whereby the heat is conveyed more quickly to the surface of the skin. At a specific moment, due to increasing loading imposed by the physical activity, the body is no longer able to shed the excess heat. Neither the muscles nor the skin can receive more blood and this is the ground for our assumption that asymmetry in the activity of muscles may result in differences in surface temperatures between the highly and less active groups of muscles; we expected this could be shown by thermal imaging. Our experiment was conducted to identify significant dependence of the measured temperature field on the surface of a selected segment (thigh muscle) with various types of loading. Similar to actual rowing in a boat, while rowing on an ergometer, the cycle of rowing comprised two phases: drive (stroke) and recovery. The drive phase includes a catch, stroke and finish. The recovery phase includes a movement forward and preparation for the next catch. Sculling involves a relatively high number of muscles, requiring intense activity of the upper and lower limbs, trunk, back and abdomen, which is not common in other sports. The symmetry in the activity of individual scullers is important for performance of rowing teams.

In literature, differences between body surfaces temperature up to 0.5°C are deemed negligible (Jones, 1998; Niu, 2001). In the case of any difference greater than 0.5°C, we may speak of asymmetry of temperature distribution, which may be the result of pathological conditions in tissues. In this study, similar to other papers on the changes in the surface temperature in response to exercise or cryogenic temperatures (Chudecka and Lubkowska, 2010, 2012; Chudecka et al., 2014), we observed no asymmetry that would exceed 0.5°C. Based on this, we concluded there was no asymmetry in the tested individuals. However, it was expected that local temperature variations would occur due to the differences in the physical activity between the studied groups of scullers and handball players. Accordingly, statistical analysis did show statistically significant differences in the group of handball players, between the left and right arm and forearm (although not exceeding the 0.5°C asymmetry threshold presented in literature). This was caused by the specificity of training in this sport, higher activity of the favored hand, for example during throws at the goal or passes.

In this context, the use of thermal imaging may be very useful for coaches, providing valuable information about the symmetry of muscle activity and thus could be used in training to improve the movement technique and motor coordination. Its advantages also include the non-invasive character, simplicity, and the short time of taking thermal images, as well as the precision of temperature measurements. In sports that use symmetric muscle activity, i.e. in two-oared sculling, it is important to engage both sides of the body in the same way in order to most effectively transform the power of the muscles into the speed of the boat, and at the same time achieve the straightest course. At the highest sporting level, this is one of the most important elements in achieving the best results.

An important aim of diagnostic tests is to identify the indicators that could help define the highest level of preparation, not only in the physical, but also in the technical skills that have a significant effect on the speed of the boat. It seems that symmetry of movement in two-oared sculling is one such an indicator, as it ensures the most economical expenditure of energy. Asymmetric activity of the human body in two-oared sculling not only adversely affects the rowing technique, but it also results in lower race times of the boat.

## Conclusions

The non-invasive and non-contact method of thermal imaging can be used to assess the activity of muscles of athletes during training, particularly in the context of a comparison of symmetric body areas.

In the group of examined scullers, during a maximal two-oared exercise on a Concept II rowing ergometer, the monitored surface temperature changes of symmetrical areas were similar, indicating that the muscle exertion was similar, especially in the context of results obtained in the group of handball players, with statistically significant differences between the temperatures of symmetrical areas of upper limbs.

## Figures and Tables

**Table 1:** Decreases in mean temperature for the right and left side of the body in a series of tests in scullers

	Right side	Left side	
	
Body area	M (SD) [ºC]	M (SD) [ºC]	Student’s t-test *p*
Arm front	3.1 (0.45)	3.1 (0.47)	0.945
Forearm front	2.1 (0.45)	2.2 (0.57)	0.802
Chest	4.3 (0.86)	4.3 (0.90)	0.927
Thigh front	2.9 (0.68)	2.8 (0.80)	0.845
Arm back	3.3 (0.85)	3.3 (0.92)	0.828
Forearm back	2.5 (0.72)	2.5 (0.72)	0.982
Back	3.8 (0.79)	3.8 (0.77)	0.984
Thigh back	2.7 (0.58)	2.6 (0.70)	0.981

**Table 2:** Decreases in mean temperature for the right and left side of the body in a series of tests in handball players

	Right side	Left side	
	
Body area	M (SD) [ºC]	M (SD) [ºC]	Student’s t-test *p*
Arm front	2.4 (0.40)	2.0 (0.39)	**0.007**
Forearm front	1.7 (0.39)	1.3 (0.38)	**0.009**
Chest	3.3 (0.57)	3.0 (0.47)	0.084
Thigh front	2.3 (0.50)	2.3 (0.48)	0.673
Arm back	2.1 (0.32)	1.8 (0.30)	**0.004**
Forearm back	1.5 (0.36)	1.1 (0.30)	**0.006**
Back	2.3 (0.47)	2.0 (0.44)	0.081
Thigh back	2.5 (0.41)	2.5 (0.43)	0.836

statistically significant differences are shown in bold (p ≤ 0.05)

## References

[b1-jhk-49-141] Akimov EB, Son’kin VD (2011). Skin temperature and lactate threshold during muscle work in athletes. Hum Physiol.

[b2-jhk-49-141] Anwajler J, Dudek K (2009). Evaluation of activity of a chosen group of muscles on the basis of temperature changes on the skin’s surface. Inż Biomed.

[b3-jhk-49-141] Aoyagi Y, McLellan TM, Shephard RJ (1997). Interactions of physical training and heat acclimation. Sports Med.

[b4-jhk-49-141] Awrejcewicz J, Byczek S, Zagrodny B (2012). Influence of the asymmetric loading of the body during the walk on the temperature distribution. Inż Biomed.

[b5-jhk-49-141] Awrejcewicz J, Zagrodny B (2011). Effect of exercise symmetry on the temperature distribution in the upper part of the human body. Inż Biomed.

[b6-jhk-49-141] Bernstein NA (1988). Exercise physiology.

[b7-jhk-49-141] Chudecka M, Lubkowska A (2010). Temperature changes of selected body’s surfaces of handball players in the course of training estimated by thermovision, and the study of the impact of physiological and morphological factors on the skin temperature. J Therm Biol.

[b8-jhk-49-141] Chudecka M, Lubkowska A (2012). The use of thermal imaging to evaluate body temperature changes of athletes during training and a study on the impact of physiological and morphological factors on skin temperature. Hum Mov.

[b9-jhk-49-141] Chudecka M, Zaborski D, Lubkowska A, Grzesiak W, Modrzejewski A, Klimek A (2014). The Study of the Dynamics of Temperature Changes in the Selected Areas of Body Surface Induced by a 10-day Program of Systemic Cryostimulation with the Use of Thermovision. Aviat Space Environ Med.

[b10-jhk-49-141] Fröhlich M, Ludwig O, Kraus S (2014). Changes in skin surface temperature during muscular endurance indicated strain - an explorative study. Int J Kinesiol Sport Sci.

[b11-jhk-49-141] Fuijmasa I (1995). Standardization of techniques for thermal imaging testing: The current situation. Biomed Thermol.

[b12-jhk-49-141] Jones BF (1998). A reappraisal of the use of infrared thermal image analysis in medicine. IEEE Trans Med Imaging.

[b13-jhk-49-141] Niu HH, Lui PW, Hu JS, Ting CK, Yin YC, Lo YL, Liu I, Lee TY (2001). Thermal symmetry of skin temperature: Normative data of normal subjects in Taiwan. Chin Med J.

[b14-jhk-49-141] Parkin S, Nowicky AV, Rutherford OM, McGregor AH (2001). Do oarsmen have asymmetries in the strength of their back and leg muscles?. J Sport Sci.

[b15-jhk-49-141] Schlader ZJ, Stannard SR, Mündel T (2010). Human thermoregulatory behavior during rest and exercise - A prospective review. Physiol Behav.

[b16-jhk-49-141] Smorawinski J, Grucza R (1994). Effect of endurance training on thermoregulatory reactions to dynamic exercise in men. Biol Sport.

[b17-jhk-49-141] Torii M, Yamasaki M, Sasaki T, Nakayama H (1992). Fall in skin temperature of exercising man. Br J Sports Med.

[b18-jhk-49-141] Vardasca R, Ring EFJ, Plassmann P, Jones CD (2012). Thermal symmetry of the upper and lower extremities in healthy subjects. Thermol Int.

